# TRA2A Binds With LncRNA MALAT1 To Promote Esophageal Cancer Progression By Regulating EZH2/β-catenin Pathway

**DOI:** 10.7150/jca.55661

**Published:** 2021-06-11

**Authors:** Xing Zhao, Qiuyang Chen, Yujie Cai, Danze Chen, Mingrong Bei, Hongyan Dong, Jianzhen Xu

**Affiliations:** 1 Computational Systems Biology Lab, Department of Bioinformatics, Shantou University Medical College (SUMC), No. 22, Xinling Road, Shantou, China.; 2 Department of Pathology and Medical Biology, University of Groningen, University Medical Center Groningen, Groningen, the Netherlands.; 3 Department of Pathology, Linyi People's Hospital, Linyi, China.

**Keywords:** TRA2A, MALAT1, Esophageal cancer

## Abstract

The RNA binding protein TRA2A, a member of the transformer 2 homolog family, plays a crucial role in the alternative splicing of pre-mRNA. However, it remains unclear whether TRA2A is involved in non-coding RNA regulation and, if so, what are the functional consequences. By analyzing expression profiling data, we found that TRA2A is highly expressed in esophageal cancer and is associated with disease-free survival and overall survival time. Subsequent gain- and loss-of-function studies demonstrated that TRA2A promotes proliferation and migration of esophageal squamous cell carcinoma and adenocarcinoma cells. RNA immunoprecipitation and RNA pull-down assay indicated that TRA2A can directly bind specific sites on MALAT1 in cells. In addition, ectopic expression or depletion of TRA2A leads to MALAT expression changes accordingly, thus modulates EZH2/β-catenin pathway. Together, these findings elucidated that TRA2A triggers carcinogenesis via MALAT1 mediated EZH2/β-catenin axis in esophageal cancer cells.

## Introduction

Esophageal cancer ranks eighth among the most common malignant tumors in the world and sixth in terms of mortality 1. There are two histological types of esophageal cancer, adenocarcinoma (EA) and squamous cell carcinoma (ESCC). The occurrence of different types of esophageal cancer has obvious regional and ethnic characteristics 2, because ESCC is more predominant in East Asia countries 3. Statistics in the past few years have shown that esophageal cancer affects the lives of more than 450,000 people around the world and its incidence is increasing rapidly year by year 4-6. The 5-year survival rate of patients with esophageal cancer is only 15-25% 7. Since it cannot be diagnosed in the early stage and has spread in the late stage, the prognosis is unfavorable 8, 9. Therefore, the identification of the key determinant and its molecular regulatory mechanisms in esophageal cancer is of great importance for the development of diagnosis biomarker and therapeutic targets.

The RNA binding protein TRA2A (Transformer 2 Alpha Homolog) is initially identified as a splicing regulator in mammalian gender determination 10, 11. In human it is located on chromosome 7 and consists of an RNA recognition motif and two arginine/serine domains located on both sides of the motif 10. In recent years, the role of TRA2A in cancer has been receiving increasing research attention. First, TRA2A is found abnormally expressed in tumors. Meta analysis of gene profiling data showed that TRA2A up-regulation was strongly associated with liver cancer 12. In pediatric pineal germinomas, TRA2A has consistent chromosomal changes, dys-regulated methylation patterns and is over-expressed in neuronal processes 13. In glioma, whole exome sequencing have shown somatic mutation of TRA2A in chemotherapy patients 14. TRA2A is high expressed in SHG44 cell to increase proliferation, migration, invasion and epithelial to mesenchymal transition 15. Second, TRA2A promotes tumorigenesis via regulating the splicing process. For example, Angulin proteins ILDR1 and ILDR2 involved in alternative pre-mRNA splicing by binding to TRA2A and other splicing factors 16. In triple negative breast cancer (TNBC), TRA2A not only facilitates TNBC cell proliferation, invasion, migration and survival, but also promotes paclitaxel resistance by regulating the alternative splicing process of RSRC2 from RSRC2s to RSRC2l 17. Finally, host TRA2A also inhibits viral mRNA splicing by binding to the intronic splicing silencer motif in the M mRNA of representative avian YS/H5N1 or in the NS mRNA of representative human PR8/H1N1 virus 18.

However, the role of TRA2A in esophageal cancer progression and the molecular regulation mechanisms have not been addressed to date. In this study, we found TRA2A binds and regulates an oncogenic lncRNA MALAT1 (Metastasis associated lung adenocarcinoma transcript 1). Genomics analysis indicates an association between TRA2A expression and disease-free survival and overall survival time in cancer patients. Functional studies also confirmed that the interaction between MALAT1 and TRA2A plays an important role in esophageal cancer cells.

## Materials and Methods

### Cell culture

ESCC cell lines TE1, EC109 were purchased from the Cell Resource Center of the Shanghai Institute of Life Sciences, Chinese Academy of Sciences (Shanghai, China). EA cell lines OE19, OE33 cell lines were from Beina Chuanglian Biotechnology (Beijing) Institute. TE1, EC109 and OE33 cell lines were cultured in RPMI 1640; OE19 cell line was maintained in Dulbecco's Modified Eagle's Medium (DMEM). All media were supplemented with 10% fetal bovine serum (FBS) and 1% penicillin/streptomycin at 37 °C and 5% CO2.

### Vector construction, oligos and transfection

Overexpression TRA2A vector EX-I0007-M14-3×Flag-TRA2A was purchased from GeneCopoeia company (Guangzhou, China). TRA2A siRNAs oligonucleotides were designed and synthesized by GenePharma (Suzhou, China). The siRNA sequences are listed in Supplementary Materials Table S1. The transfections of vectors and siRNAs were performed using Lipofectamine 3000 (Invitrogen, USA). All experiments were conducted according to the manufacturer's recommendations.

### RNA isolation and RT-qPCR

Total RNA was isolated using TRIzol reagent (Invitrogen, Carlsbad, CA, USA) following the manufacturer's procedure. Reverse Transcription and RT-qPCR quantification were performed using PrimeScriptTM RT reagent kit with gDNA Eraser (RR470A, Takara) and TB GreenTM Premix Ex TaqTM II (RR820A, Takara), respectively, according to the manufacturer's protocol. Relative gene expression was calculated by using the 2^-ΔΔCt^. The primers are listed in Supplementary Materials Table S2.

### Proliferation and migration assay

Cell Counting Kit-8 assay was used to detect cell proliferation. In total of 100 μl and 2 ×10^3^ cells were seeded into 96-well plates. Then, 10 μl of CCK-8 solution (Beyotime, Shanghai, China) was added to each well on days 1, 2, 3, 4 and 5. After 2 h of incubation in the cell incubator, the absorbance at 450 nM was measured. For colony formation assay, 400 cells were plated in 6-well plates and incubated at 37°C in a 5% CO2 for about two weeks until the colonies are visible. The colonies were fixed with methanol solution for 15 minutes, stained with 0.1% crystal violet for 30 minutes, and counted after cleaning. For the transwell migration assay, 1×10^4^ cells were seeded in the upper transwell chamber (8 μm pore size, Corning, NY, USA) in a volume of 200 μl medium without FBS, while 600μl medium with 10% FBS was added to the 24-well plate with transwell chamber. After 48h culture in the incubator, the cells in the upper transwell chamber were gently wiped off, and the cells attached to the outside of the filter were fixed with methanol and stained with 0.1% crystal violet. For would healing assay, the monolayer was scratched using a sterile 200 μl pipette tip after serum starvation for 12h when the cell confluence was close to 100%. Then the cells were cultured in complete medium after detached cells were removed. Photos were taken at 0, 12 and 24h. Wound closure rate was calculated as follows: wound closure rate (%) = (initial wound distance - final wound distance)/initial wound distance ×100.

### RNA immunoprecipitation (RIP) assays

RIP assays were performed in TE1 cells transfected TRA2A vector 48h, using Magna RIP RNA-Binding Protein Immuno-precipitation Kit (Millipore, USA) according to the manufacturer's instructions. First, cells were washed in cold PBS and resuspended in RIP Lysis Buffer combined with a protease inhibitor cocktail and RNase inhibitors. Then 100μl cell lysates were incubated with beads coated with 5 μg of control mouse IgG or antibody against Flag (Santa cruz, USA) with rotating overnight at 4 °C. After the lysates were treated with proteinase K buffer, immunoprecipitated RNA was extracted and reverse transcribed using PrimeScriptTM RT reagent kit with gDNA Eraser (RR470A, Takara). The expression of MALAT1 was tested by RT-qPCR.

### RNA pull-down and iTRAQ assays

The MALAT1 sequence (NR_002819.3) was divided into four fragments (F1 nts 1-2340; F2 nts 2341-4500; F3 nts 4501-6301; F4 nts 6302-8751) and cloned into the pEASY-blunt vector with the T7 promoter. Each fragment is ~2000bp, avoiding the predicted protein binding domains. The primers to construct MALAT1 are listed in Supplementary Materials Table S3. After linearization of plasmids, T7 RNA polymerase and biotin-labeled mixture were used to transcribe RNA *in vitro*. The labeled RNA was bound to magnetic beads and then incubated with cell lysates to get RNA&Protein complex. After eluting the RNA&Protein complex, proteins associated with biotin-labeled RNAs were immunoprecipitated with streptavidin magnetic beads, and subjected to perform iTRAQ (isobaric tags for relative and absolute quantitation) experiment using AB SCIEX TripleTOF^®^ 6600 System and related AB SCIEX ProteinPilot™ 4.5 software.

### Western blot

Cells were placed on ice for 30 min in a RIPA lysis buffer and PMSF (Solarbio, Beijing, China). After protein extraction, 30 μg protein per sample was loaded to 10% SDS-polyacrylamide gel electrophoresis (SDS-PAGE) and transferred to polyvinylidene fluoride membranes (Immobilon P, Millipore, Billerica, USA). After blocking with 5% non-fat milk, the membranes were incubated with primary antibody at 4°C overnight, followed by incubation with secondary antibodies at room temperature for 1 h. The antibodies including GAPDH (#2118, 1:1000), EZH2 (#5246, 1:1000) and β-catenin (#8480, 1:1000) were purchased from Cell Signaling Technology (USA) and TRA2A (ab72625, 1:1000) was bought from Abcam.

### Statistical analysis

Data were collected and expressed as the mean ± standard error of the mean (S.E.M.) and were analysed using SPSS 19.0 (IBM, Armonk, NY, USA) with differences between two groups assessed by independent T test. Statistical probability of P < 0.05 was considered significant. Graphs were generated with GraphPad Prism 7.0.

## Results

### TRA2A expression characteristics in esophageal cancer

**Figure 1:**
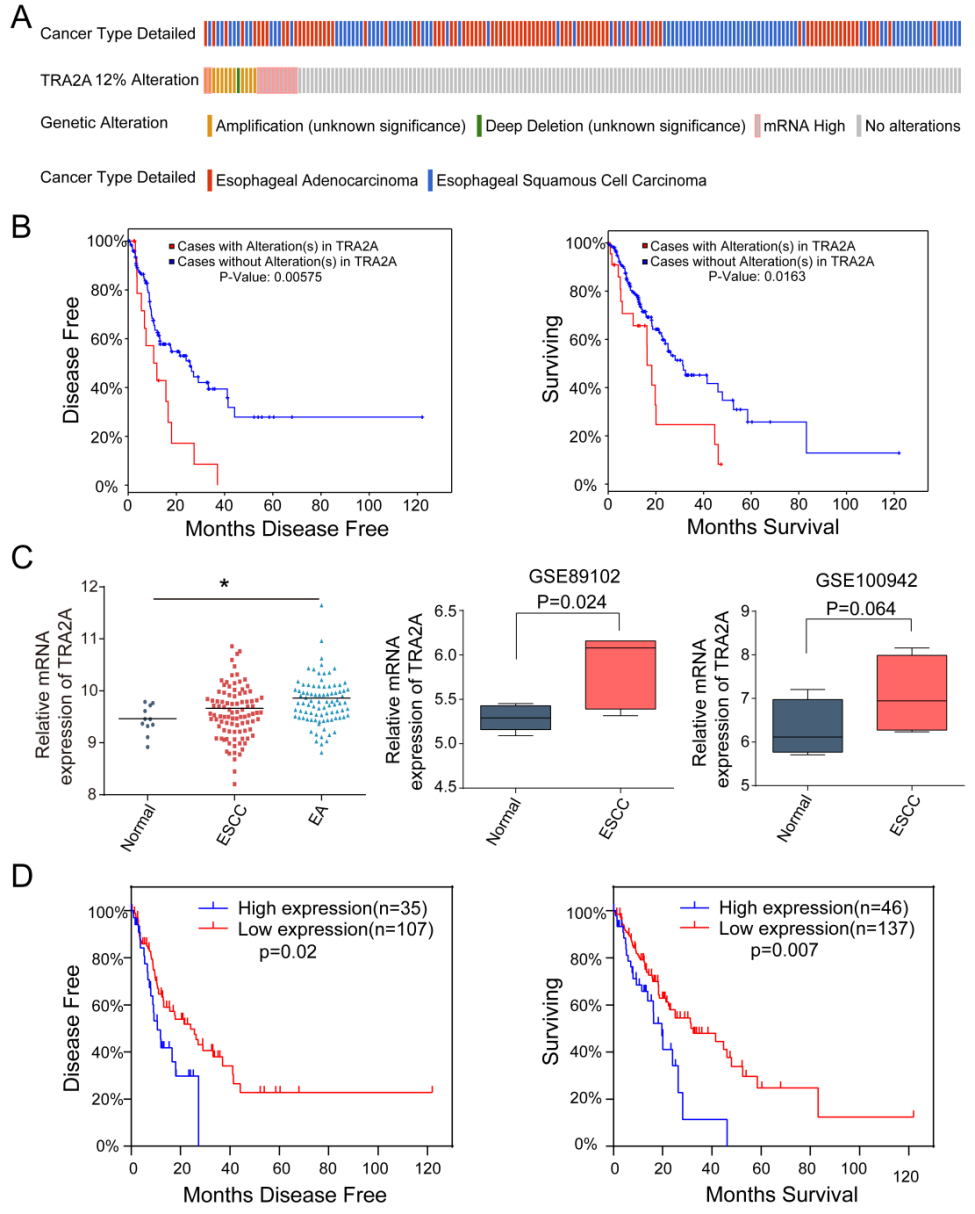
TRA2A expression characteristics in esophageal cancer. **(A)** Overview of TRA2A genetic alteration in 184 EC samples; **(B)** Kaplan-Meier plots showing the association between TRA2A genetic alteration and either disease-free survival or overall survival in 184 EC samples; **(C)** TRA2A mRNA expression in 194 TCGA esophageal samples (left) and the two GEO gene expression profiles (right). Each point represents one tissue sample; **(D)** Kaplan-Meier plots showing the association between TRA2A mRNA expression and either disease-free survival or overall survival in 184 EC samples. ^*^ P≤0.05.

Currently there are few reports on TRA2A expression characteristics in human cancer cells. So we first evaluated the mRNA expression profiles of TRA2A based on Cancer Cell Line Encyclopedia (CCLE) (https://portals.broadinstitute.org/ccle). TRA2A is shown to express at stable level in a variety of cancer cell lines (Supplementary Materials Figure S1A). It is also expressed in all 27 measured esophageal cancer cell lines (Supplementary Materials Figure S1B). We further downloaded and analyzed 194 esophageal samples from The Cancer Genome Atlas (TCGA) (https://www.cancer.gov/about-nci/organization/ccg/research/structural-genomics/tcga), of which 10 are normal, 95 are ESCC and 89 are EAC. Approximately 12% of tumor samples were shown different type of genetic alteration including amplification (12 cases), deep deletion (1 case), and mRNA upregulation (12 cases) (Figure 1A). Patients with TRA2A alteration are significantly correlated with shorter disease-free survival (*P* =0.006) and overall survival time (*P* =0.016) (Figure 1B), indicating the clinical relevance of TRA2A genetic alterations.

Focusing on mRNA expression, we observed increased TRA2A in both ESCC and EA samples. Two additional datasets from Gene Expression Omnibus (GEO: https://www.ncbi.nlm.nih.gov/geoprofiles/) validated the up-regulation of TRA2A in ESCC samples *vs* matched nonmalignant esophageal samples (Figure 1C). High TRA2A expression levels had shorter disease-free survival (*P* =0.02) and overall survival time (*P* =0.007) (Figure 1D). Together, above results suggested that TRA2A is abnormally up-regulated in esophageal cancer.

### TRA2A regulates EC cells proliferation and migration

**Figure 2:**
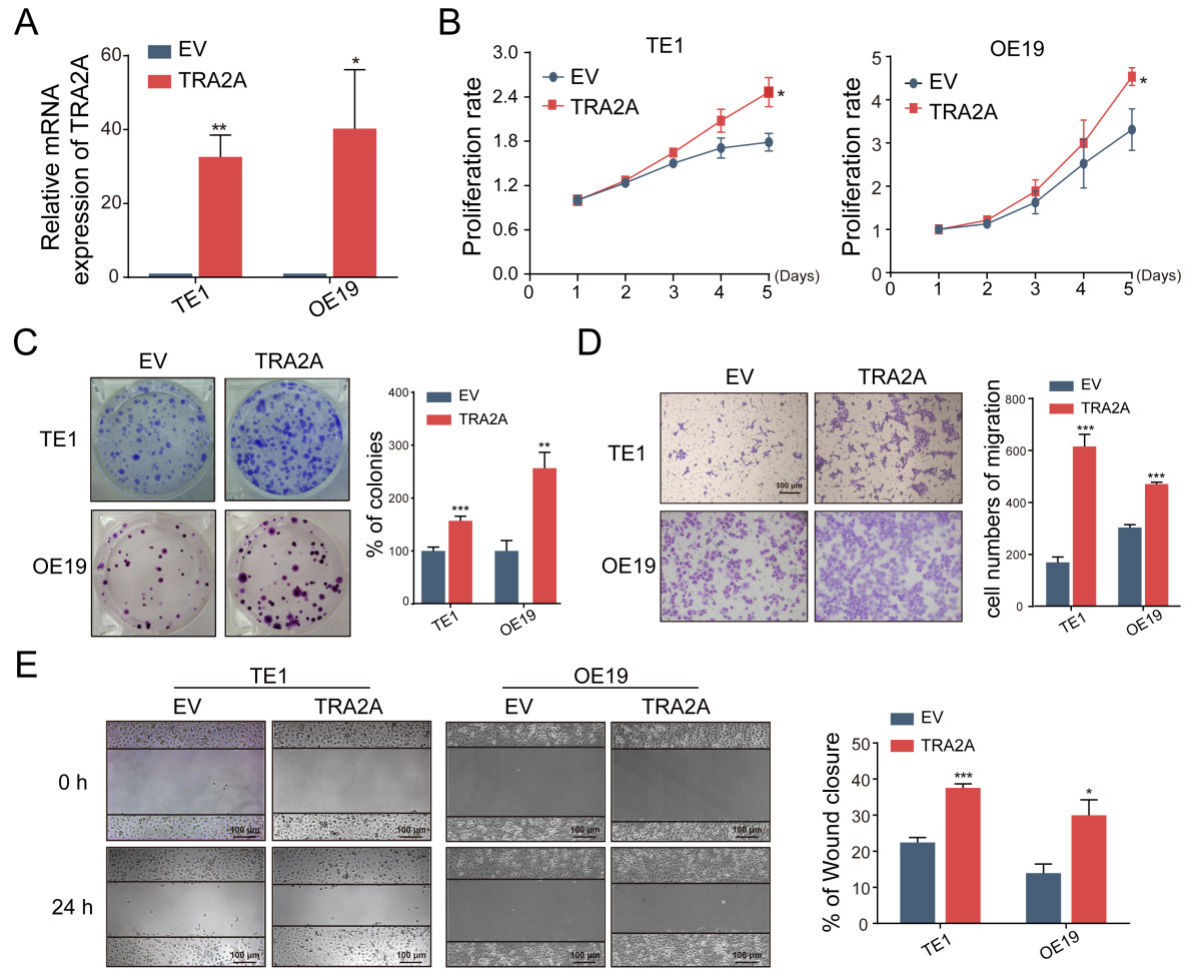
Up-regulated TRA2A enhances EC cells proliferation and migration. **(A)** qRT-PCR analysis validating TRA2A mRNA expression after transfecting overexpression vector; **(B)** CCK8 assays; **(C)** Colony formation assays; **(D)** Transwell migration assays and **(E)** Wound healing assays were performed to determine the effect of TRA2A on cell proliferation and migration. EV, empty vector. Mean ± SEM are shown, n = 3. ^*^ *P*≤0.05; ^* *^ *P*≤0.01; ^* * *^ *P*≤0.001.

**Figure 3:**
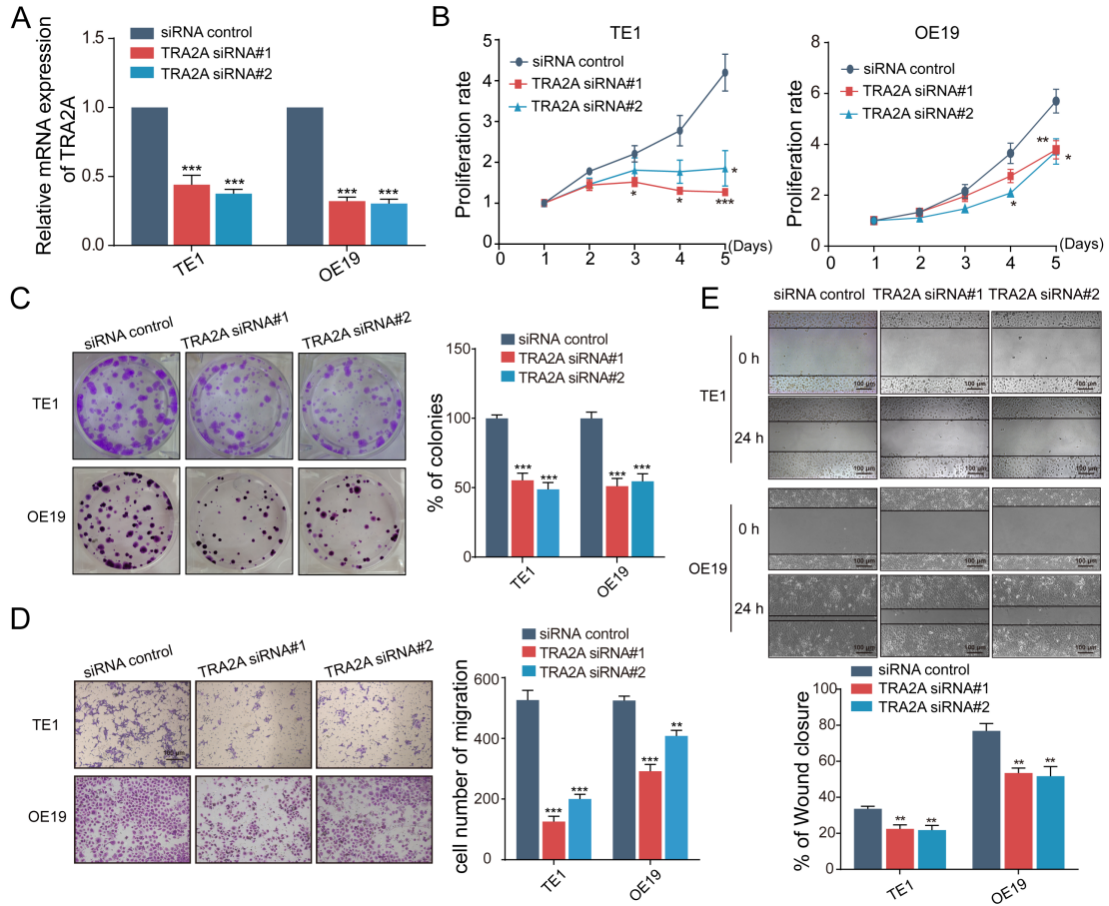
Reduced TRA2A inhibits EC cells proliferation and migration. **(A)** qRT-PCR analysis confirming TRA2A mRNA expression after transfecting TRA2A siRNAs; **(B)** CCK8 assays; **(C)** Colony formation assays; **(D)** Transwell migration assays and **(E)** Wound healing assays were performed to determine the effect of TRA2A depletion on cell proliferation and migration. Mean ± SEM are shown, n = 3. ^*^ *P*≤0.05; ^* *^ *P*≤0.01; ^* * *^ *P*≤0.001.

To investigate the potential function of TRA2A in esophageal carcinoma, we constructed TRA2A over-expression vector and tested TRA2A expression efficiency in both esophageal squamous cell carcinoma cell TE1 and esophageal adenocarcinoma cell OE19 (Figure 2A). Up-regulated TRA2A could promote proliferation and migration in both TE1 and OE19 cells (Figure 2B-D) as demonstrated by gain-of-function approaches including CCK8, colony formation, transwell and wound healing. Two specific siRNAs were designed to efficiently knock down TRA2A in TE1 and OE19 cell lines (Figure 3A). Importantly, silencing of TRA2A greatly reduced proliferation, colony formation and migration ability in esophageal cell lines (Figure 3B-D). Collectively, these data confirmed that TRA2A is involved in proliferation and migration of EC cells.

### TRA2A binds and regulates lncRNA MALAT1

Previously, we have developed a computational pipeline to find the interactions among RNA binding protein, lncRNA and miRNA 19. Our predictions indicated that TRA2A binds with lncRNA MALAT1 in esophageal cells. ENCODE eCLIP data (https://www.encodeproject.org/) also showed that MALAT1 is one of the 108 overlapped RNAs binding with TRA2A in both HepG2 and K562 cells (Supplementary Materials Figure S2A).

**Figure 4:**
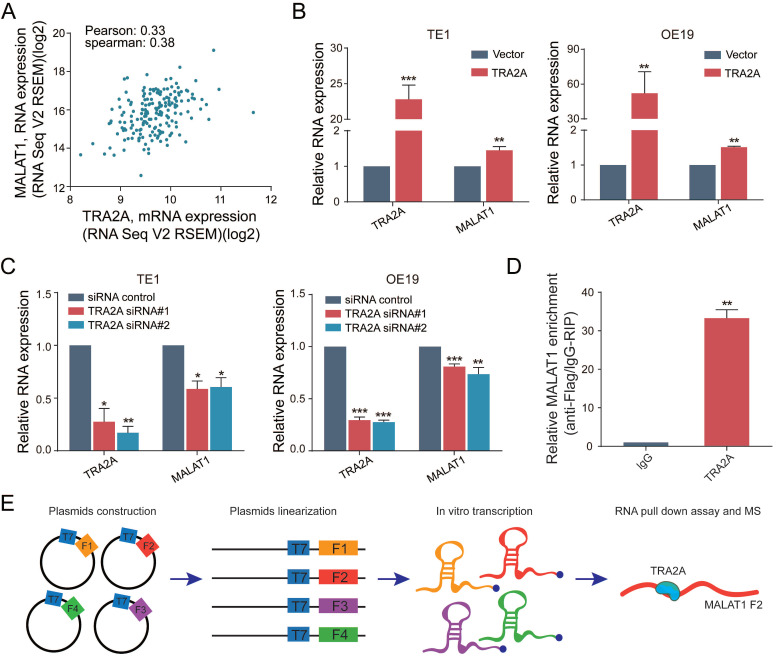
TRA2A binds and regulates lncRNA MALAT1. **(A)** Correlation analysis between TRA2A mRNA expression and MALAT1 RNA expression in 184 EC samples from TCGA; **(B)** RNA expression levels of TRA2A and MALAT1 after overexpression of TRA2A;** (C)** RNA expression levels of TRA2A and MALAT1 after transfecting TRA2A siRNAs; **(D)** TRA2A overexpression plasmid with flag label was transfected into TE1 cells, MALAT1 expression was detected after RIP using flag antibody; **(E)** A schematic figure shown the RNA pull down assay and mass spectrometric result. Mean ± SEM are shown, n = 3. ^*^ *P*≤0.05; ^* *^ *P*≤0.01; ^* * *^ *P*≤0.001.

To further explore the relationship between TRA2A and MALAT1, we analyzed the RNA expression correlation between TRA2A and MALAT1 in 184 esophageal cancer samples from TCGA. The results showed that TRA2A was positively correlated with MALAT1 expression in EC samples (Pearson correlation coefficient= 0.33, spearman correlation coefficient= 0.38) (Figure 4A). When samples were analyzed separately for ESCC and EA, TRA2A was found to have a higher positive correlation with MALAT1 expression in EA (Pearson correlation coefficient=0.44, correlation coefficient= 0.38, Supplementary Materials Figure S2C) than in ESCC (Pearson correlation coefficient=0.18, spearman correlation coefficient= 0.28, Supplementary Materials Figure S2B). Supporting above analysis, MALAT1 was increased after overexpression of TRA2A in TE1 and OE19 cell lines (Figure 4B), which was also observed in another two esophageal cell lines EC109 and OE33 (Supplementary Materials Figure S2D). Moreover, we also detected reduced MALAT1 expression after silencing TRA2A (Figure 4C and Supplementary Materials Figure S2E). All these results indicated that the expression of MALAT1 is regulated by TRA2A.

Besides, RT-qPCR analysis after RNA immunoprecipitation assays in TE1 cells using flag antibody confirmed an enrichment of MALAT1, compared with IgG control (Figure 4D). We used RBPmap (http://rbpmap.technion.ac.il/) to predict the potential binding sites of TRA2A on MALAT1 (Supplementary Materials Table S4). Next, the whole sequence of MALAT1 was divided into four fragments and transcribed *in vitro*. Then the proteins associated with biotin-labeled RNAs were pull down and subjected to mass spectrometric analysis (Figure 4E). The results showed that TRA2A binds to the fragment 2 of MALAT1 (Supplementary Materials File 1), consistent with the predicted results of RBPmap. Taken together, these results suggested that TRA2A can directly bind with MALAT1, and regulate the lncRNA expression in esophageal cancer cells.

### TRA2A modulates EZH2 pathway by binding with MALAT1

**Figure 5:**
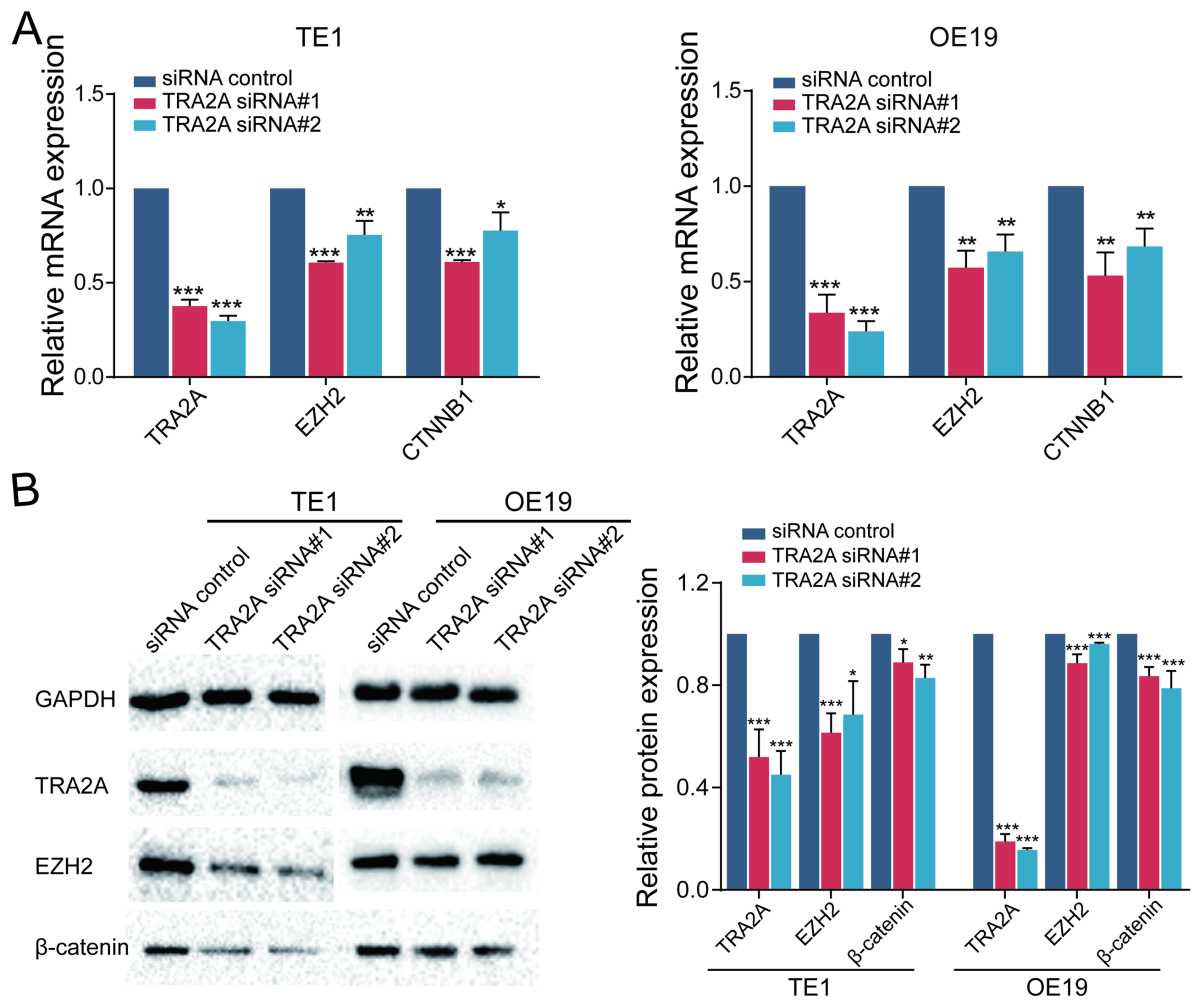
TRA2A modulates EZH2 pathway. **(A)** RNA expression levels of TRA2A, EZH2 and CTNNB1 after transfecting TRA2A siRNAs; **(B)** Protein expression of TRA2A, EZH2 and β-catenin after silencing TRA2A. Left panel, representative blots. Right panel, quantification of protein expression was normalized to GAPDH and the ratio of TRA2A to GAPDH was treated as 1. Mean ± SEM are shown, n = 3. * P≤0.05; * * *P*≤0.01; * * * P≤0.001.

MALAT1 is known to promote esophageal cancer progression 20, 21. In particular, MALAT1 can activate EZH2 pathway in aggressive renal cell carcinoma, colorectal cancers and osteosarcoma 22-24. Thus we postulated that TRA2A regulates and activates the EZH2/β-catenin pathway by binding to MALAT1 and elevating its expression, hence promotes esophageal cancer progression. To address this hypothesis, we performed RT-qPCR after inhibition of TRA2A with siRNAs, which revealed that TRA2A reduces EZH2 and CTNNB1 (β-catenin) in both TE1 and OE19 (Figure 5A). In addition, proteins expressions of TRA2A, EZH2 and CTNNB1 (β-catenin) were tested after TRA2A knockdown. As shown in Figure 5B, expression of EZH2, CTNNB1 (β-catenin), were significantly decreased in TE1 after depletion of TRA2A by small interfering RNAs. Similar results were also observed in another esophageal cancer cell OE19 (Figure 5B). Together, these data suggested that binding of TRA2A to MALAT1 activates EZH2/β-catenin pathway.

## Discussion

Increasing evidences indicated long noncoding RNAs (LncRNAs) play a fundamental regulatory roles in esophageal cancer progression 25. MALAT1 is a highly expressed lncRNA in EC tissues and correlated with lymphatic invasion, distant metastasis and tumor differentiation nuclear lncRNA 26, 27. Several mechanisms of action of MALAT1 in esophageal squamous cell carcinoma have been elucidated. Firstly, MALAT1 sponges miRNAs such as miR-101 and miR-217, which leads to the decrease of miRNA target mRNAs 21. MALAT1 also recruits EZH2 (enhancer of zeste 2 polycomb repressive complex 2 subunit) to form the PRC2 complex followed by transcriptional repression of the target loci 20, 24. Since MALTAT1 is a large, 8758bp-long lncRNA and harbors multiple protein binding sites, novel molecular mechanisms may exist in ESCC 25.

Here, we have shown that TRA2A was directly bound to MALAT1 via RIP assay. Using a complementary RNA pull down experiments, we confirmed that TRA2A binds to the 2341-4500 bp fragment of MALAT1. Furthermore, TRA2A influences the expression of MALAT1. We speculated that TRA2A interaction with MALAT1 may enhance the lncRNA stability in EC. Indeed, another lncRNA, LINC00662, was recently reported to bind with TRA2A. This interaction increases LINC00662 stability, thereby to regulate blood-brain barrier permeability in the Alzheimer's microenvironment 28. In addition, we have demonstrated that the binding of TRA2A to MALAT1 controls the EZH2/β-catenin pathway in siRNA depletion assay. Recently, the regulatory role of TRA2A in ncRNA m6A modification has been suggested 29. Thus TRA2A may selectively promote the methylations of the m6A sites co-localized with its binding sites on MALAT1 through physical interactions with the m6A writers. This methylation will lead to MALAT1 structure change, which further facilitates PRC2/EZH2 histone methylase complex formation. But the specific regulation mechanism needs more studies. Functionally, we demonstrated that TRA2A is associated with proliferation and migration of esophageal cancer. We also studied the expression characteristics of TRA2A among esophageal cancer patients. TRA2A was up-regulated in esophageal cancer compared to normal tissues, and patients with high TRA2A expression had shorter disease-free survival and overall survival time.

In summary, this study demonstrates an important regulatory role of TRA2A-MALAT1 interaction in esophageal cancer cells. In addition to its canonical function as a mRNA splicing factor, TRA2A directly binds to lncRNA as an RNA-binding protein and participates in cancer signaling transduction, therefore providing new insights into mechanism and treatment.

## Supplementary Material

Supplementary figures and tables.

## Abbreviations

TRA2A: Transformer 2 Alpha Homolog
MALAT1: Metastasis associated lung adenocarcinoma transcript 1
EZH2: Enhancer of zeste 2 polycomb repressive complex 2 subunit
TCGA: The Cancer Genome Atlas
GEO: Gene Expression Omnibus
RIP: RNA immunoprecipitation
