# Supplementary material for: TRA2A Binds With LncRNA MALAT1 To Promote Esophageal Cancer Progression By Regulating EZH2/β-catenin Pathway
Source: J Cancer. 2021 Jun 11;12(16):4883–90. doi: 10.7150/jca.55661 (PMC8247389; doi:10.7150/jca.55661)
Supplement: Supplementary file 1 — Supplementary figures and tables. [file jcav12p4883s1.pdf]

Supplementary Materials

Table S1. siRNA sequences.

Name	Sense (5'-3')	Antisense (5'-3')
siRNA control	UUCUCCGAACGUGUCACGUTT	ACGUGACACGUUCGGAGAATT
TRA2A siRNA#1	GGGCAAAUCCAGAUCCCAATT	UUGGGAUCUGGAUUUGCCCTT
TRA2A siRNA#2	GCCUCAGUUUGUACACAACTT	GUUGUGUACAAACUGAGGCTT

Table S2. RT-qPCR primer sequences.

Name		sequence（5′-3′）	Product size
β-actin	Forward	CATGTACGTTGCTATCCAGGC	250 bp
	Reverse	CTCCTTAATGTCACGCACGAT	
TRA2A	Forward	CTCCAATGTCTAACCGGAGAAGA	148 bp
	Reverse	TTGACACCACTCAATGGTCCA	
MALAT1	Forward	GACGGAGGTTGAGATGAAGC	84 bp
	Reverse	ATTCGGGGCTCTGTAGTCCT	
EZH2	Forward	GGACCACAGTGTTACCAGCAT	79 bp
	Reverse	GTGGGGTCTTTATCCGCTCAG	
CTNNB1	Forward	AGCTTCCAGACACGCTATCAT	98 bp
	Reverse	CGGTACAACGAGCTGTTTCTAC	

Table S3. Primer sequences to construct overexpressed MALATA vector for pull down assay.

Name	sequenses（5′-3′）
MALAT1-F1 Forward	ATTTGCGGCCGCGTAAAGGACTGGGGCCCCGC
MALAT1-F1 Reverse	CCGCTCGAGCTGGTCTACGTAAACACCCTCATCT
MALAT1-F2 Forward	ATTTGCGGCCGCAACCAATTTAGAAGAATACTTGAAG
MALAT1-F2 Reverse	CCGCTCGAGCACTACCATATCCAAACAACTTTTG
MALAT1-F3 Forward	ATTTGCGGCCGCTGTGGTTCTCTTTTGGAATTTTTTT
MALAT1-F3 Reverse	CCGCTCGAGCATTGCCTACCACTCTAAGATTGTA
MALAT1-F4 Forward	ATTTGCGGCCGCTTTACACTATTGACCTTATATAGGG
MALAT1-F4 Reverse	CCGCTCGAGTTCCCCAATCAAGATTTTTTTATTC

Table S4. Predicted binding sites of TRA2A on MALAT1 by RBPmap.

Sequence	Genomic position	Motif	K-mer	Z-score	P-value
1471	chr11:65499232	gaagagga	ag uaagagaaaa	2.62	4.40E-03
1478	chr11:65499239	gaagagga	ag aaauaugaag	3.13	8.74E-04
1484	chr11:65499245	gaagagga	ag gaagacuuag	3.25	5.77E-04
1493	chr11:65499254	gaagagga	ag gaagaguagc	3.25	5.77E-04
1502	chr11:65499263	gaagagga	ag caugaggaag	3.402	3.34E-04
1508	chr11:65499269	gaagagga	ag gaaggaaaag	2.891	1.92E-03
1894	chr11:65499655	gaagagga	ag gaaguggaaa	2.685	3.63E-03
1902	chr11:65499663	gaagagga	ag aaacuggaag	3.033	1.21E-03
1918	chr11:65499679	gaagagga	ag guacgggaag	3.033	1.21E-03
1924	chr11:65499685	gaagagga	ag gaaggcgaag	2.685	3.63E-03
1982	chr11:65499743	gaagagga	ag gaagaaaaaa	2.141	1.61E-02
2007	chr11:65499768	gaagagga	ag gaaaaguagg	3.25	5.77E-04
2010	chr11:65499771	gaagagga	ag aaguaggaag	3.13	8.74E-04
2016	chr11:65499777	gaagagga	ag gaagcagaag	3.402	3.34E-04
2019	chr11:65499780	gaagagga	ag gcagaagaaa	3.13	8.74E-04
2022	chr11:65499783	gaagagga	ag gaagaaaaaa	3.13	8.74E-04
2299	chr11:65500060	gaagagga	ag gaagguaaag	2.424	7.68E-03
2314	chr11:65500075	gaagagga	ag gaagaugagg	2.685	3.63E-03
2317	chr11:65500078	gaagagga	ag gaugagggug	2.424	7.68E-03
2521	chr11:65500282	gaagagga	ag guaacggaag	2.141	1.61E-02
2536	chr11:65500297	gaagagga	ag caagaucaag	2.141	1.61E-02
2542	chr11:65500303	gaagagga	ag caagaguaau	2.141	1.61E-02
2792	chr11:65500553	gaagagga	ag gugaaggaag	2.62	4.40E-03
2803	chr11:65500564	gaagagga	ag uaggaagaag	2.62	4.40E-03
2806	chr11:65500567	gaagagga	ag gaagaaggaa	2.62	4.40E-03
2807	chr11:65500568	gaagagga	ag aagaaggaag	2.62	4.40E-03
4570	chr11:65502331	gaagagga	ag gcaaaggaag	1.739	4.10E-02
4842	chr11:65502603	gaagagga	ag gaugagggag	1.739	4.10E-02
5177	chr11:65502938	gaagagga	ag gaagaggcaa	1.739	4.10E-02
7699	chr11:65505460	gaagagga	ag gaaggggagg	1.739	4.10E-02

Notes. The list is all the predicted TRA2A binding sites on MALAT1. The binding sites on fragment 2 (nts 2341-4500) of MALAT1 are labeled in blue.

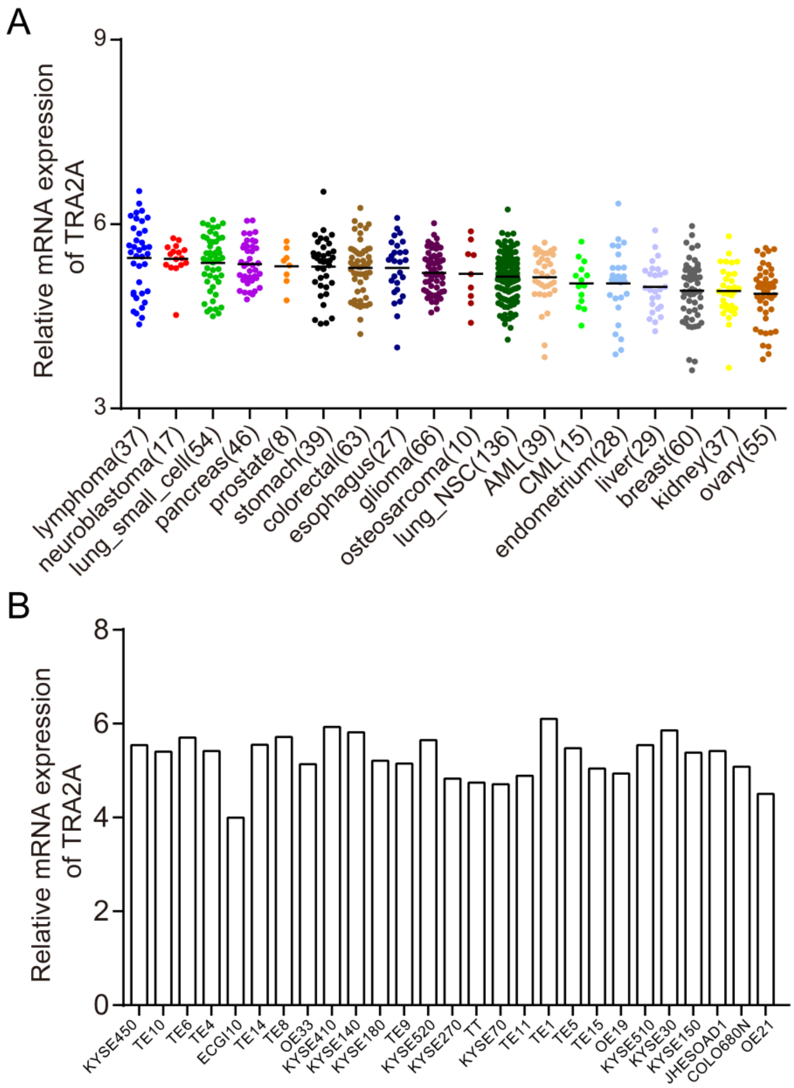

Figure S1. (**A**) TRA2A mRNA expression in different cancer cells from CCLE. Each point represents one cell line. Black line means average expression level; (**B**) TRA2A mRNA expression in different esophagus cancer cell lines from CCLE.

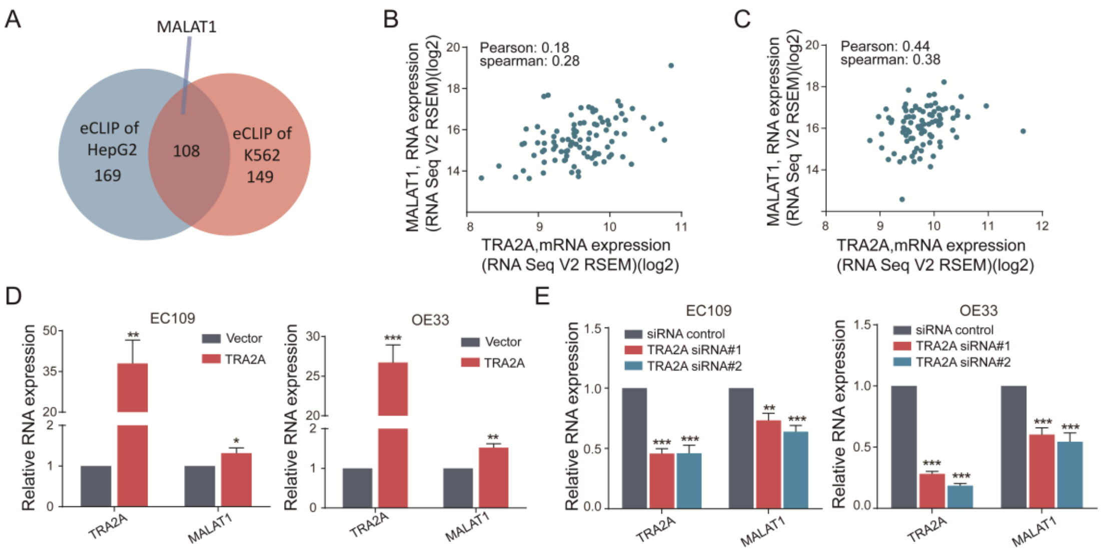

Figure S2. Correlation between TRA2A and MALATA RNA expression. (**A**) Overview of eCLIP data analysis from ENCODE; (**B-C**) Correlation analysis between TRA2A mRNA expression and MALAT1 RNA expression in 95 TCGA ESCC samples (B) and 89 TCGA EA samples (**C**). Every point represents one tissue sample; (**D-E**) RNA expression levels of TRA2A and MALAT1 after overexpression of TRA2A (**D**) or transfecting TRA2A siRNAs (**E**) in EC109 and OE33 cell lines. Mean ± SEM are shown, n = 3. * *P*≤0.05; ** *P*≤0.01; *** *P*≤0.001.
